# Comparison of autologous hematopoietic cell transplantation, matched sibling donor hematopoietic cell transplantation, and chemotherapy in patients with favorable- and intermediate-risk acute myeloid leukemia

**DOI:** 10.3389/fimmu.2024.1511057

**Published:** 2025-01-08

**Authors:** Mingyang Wang, Shulian Chen, Qiuqiu Zhang, Linyu Yuan, Xue Wang, Junshi Zhang, Xiaoyu Zhang, Yigeng Cao, Dongmei Li, Xinxiao Lu, Meijiao Wang, Xiaosi Jiang, Rongli Zhang, Xin Chen, Qiaoling Ma, Jialin Wei, Donglin Yang, Yi He, Aiming Pang, Sizhou Feng, Mingzhe Han, Weihua Zhai, Xingli Zhao, Erlie Jiang

**Affiliations:** ^1^ State Key Laboratory of Experimental Hematology, National Clinical Research Center for Blood Diseases, Haihe Laboratory of Cell Ecosystem, Institute of Hematology and Blood Diseases Hospital, Chinese Academy of Medical Sciences and Peking Union Medical College, Tianjin, China; ^2^ Department of Hematology, Oncology Center, Tianjin Union Medical Center of Nankai University, Tianjin, China; ^3^ College of Pharmacy, Nankai University, Tianjin, China

**Keywords:** acute myeloid leukemia, favorable- and intermediate-risk, hematopoietic stem cell transplantation, chemotherapy, post-remission treatment

## Abstract

**Introduction:**

Hematopoietic stem cell transplantation (HSCT) and chemotherapy are considered potentially curative options for post-remission therapy in acute myeloid leukemia (AML). However, the comparative effectiveness of these approaches in favorable- and intermediate-risk AML remains unclear and requires further investigation.

**Methods:**

In this retrospective study, 111 patients diagnosed with de novo favorable- and intermediate-risk AML, categorized according to the ELN 2022 guidelines, were investigated to compare outcomes following autologous HSCT (auto-HSCT), matched sibling donor HSCT (MSD-HSCT), and chemotherapy. Through propensity score matching for disease status before HSCT, 42 cases in first complete remission were selected for each of the auto-HSCT group and the MSD-HSCT group. Additionally, 27 cases in the chemotherapy group, excluding patients with early relapse or death, were included for comparison.

**Results:**

In the overall population, the 3-year overall survival (OS) rates were 85.7%, 83.1%, and 70.4% (p = 0.043), while the disease-free survival (DFS) rates were 78.6%, 83.2%, and 57.1% (p = 0.002) in the auto-HSCT, MSD-HSCT, and chemotherapy groups, respectively. Notably, both auto-HSCT and MSD-HSCT demonstrated significantly improved DFS compared to chemotherapy in patients with favorable-risk AML. Multivariate analysis further revealed that chemotherapy was significantly associated with inferior DFS compared to auto-HSCT (HR=2.82; 95% CI, 1.26–6.32, p=0.012), while DFS was similar between the MSD-HSCT and auto-HSCT groups (HR=0.80; 95% CI, 0.31–2.09, p=0.645).

**Discussion:**

The findings suggested the advantages of both MSD-HSCT and auto-HSCT over chemotherapy as post-remission therapy for AML patients with favorable and intermediate risk. Further research is needed to support these conclusions.

## 1 Introduction

Acute myeloid leukemia (AML) is the most common acute leukemia in adults; after complete remission (CR) was achieved with “3 + 7” chemotherapy or new agents, subsequent treatment needed to be selected. Currently, consolidation strategies are chemotherapy and autologous (auto) and allogeneic (allo) hematopoietic stem cell transplantation (HSCT). Treatment decision is mainly based on cytogenetic risk stratification and dynamic Minimal Residual Disease (MRD) monitoring. Allo-HSCT is recommended for high-risk AML in patients (1–3), but there are different recommendations of post-remission options for favorable- and intermediate-risk patients. The study by Koreth suggested that allo-HSCT does not provide significant benefit for favorable-risk AML (4). A study by Lv found that compared with chemotherapy alone, haplo-HSCT confers significant survival advantages in terms of disease-free survival (DFS), overall survival (OS), and cumulative incidence of relapse (CIR) for patients with intermediate-risk AML in first CR (CR1) (5), while another study also confirmed that matched sibling donor HSCT (MSD‐HSCT) was associated with lower CIR and increased OS as compared to auto-HSCT in intermediate-risk AML (6). Currently, many studies have confirmed the importance of auto-HSCT for a subset of AML patients. The study by Wang provided evidence for the use of auto‐HSCT as a viable therapeutic option for favorable‐ and intermediate‐risk AML patients in CR1 with persistent undetectable MRD (uMRD) (7).

This retrospective study was planned to compare the efficacy of chemotherapy, auto-HSCT, and MSD‐HSCT in AML patients with favorable- and intermediate-risk AML who were in CR1.

## 2 Methods

### 2.1 Study design and data collection

**Figure 1:**
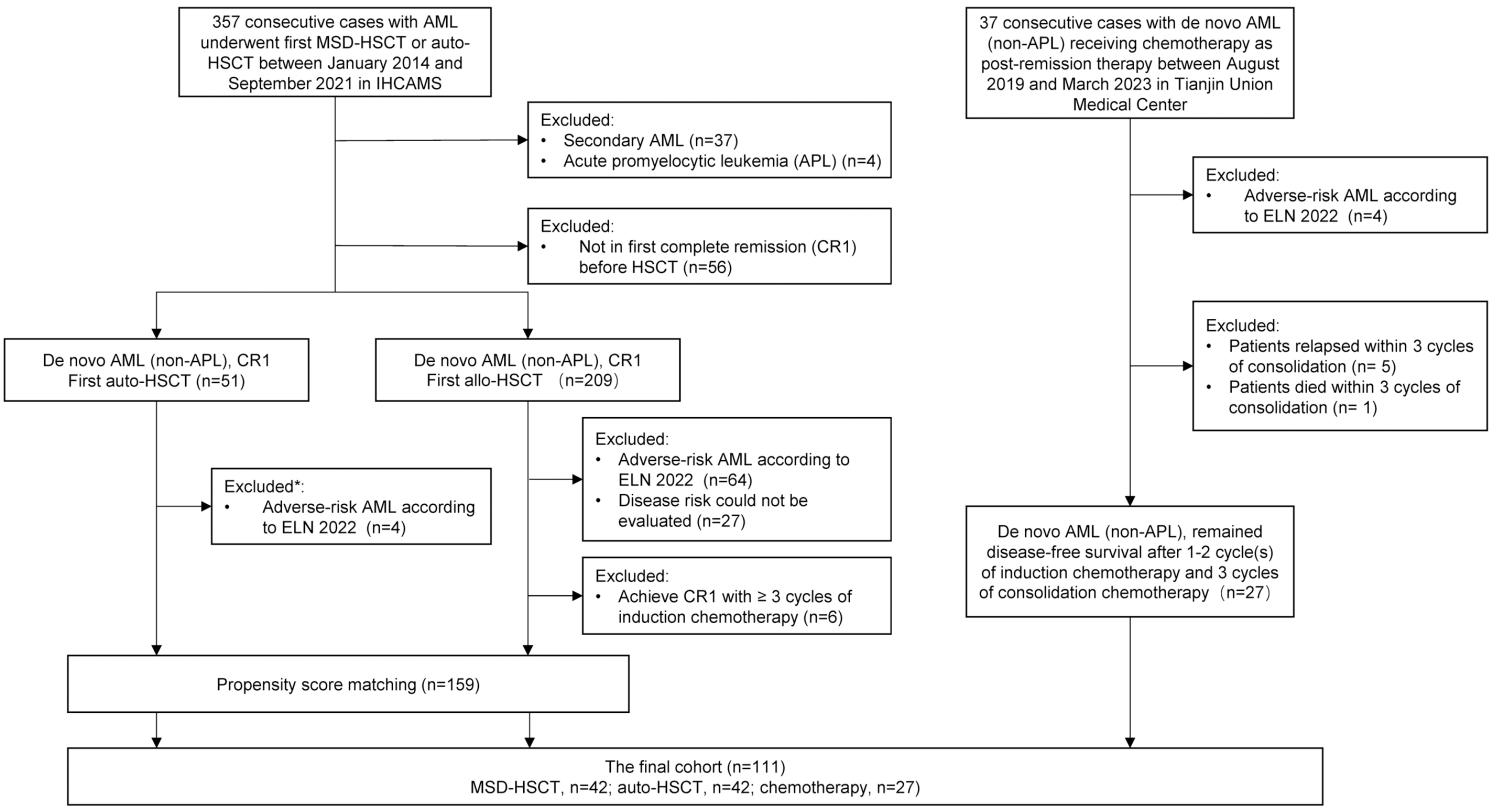
Flowchart for patients’ selection. *25 of 51 (49.0%) patients could not accurately assess disease risk according to ELN 2022 because the panel of gene mutation did not cover ASXL1, BCOR, EZH2, RUNX1, SF3B1, SRSF2, STAG2, U2AF1, and ZRSR2. Thirteen out of 25 (52%) patients with favorable genetic abnormalities were classified as favorable-risk, while the remaining 12 patients (48%), who did not show evidence of adverse genetic abnormalities, were classified as intermediate-risk. AML, acute myeloid leukemia; HSCT, hematopoietic stem cell transplantation; MSD-HSCT, matched sibling donor HSCT; auto-HSCT, autologous HSCT; IHCAMS, Institute of Hematology, Chinese Academy of Medical Sciences. CR1, first complete remission.

This was a retrospective comparative analysis, comparing auto-HSCT and MSD-HSCT to chemotherapy for favorable- and intermediate-risk *de novo* AML based on the 2022 ELN criteria (8). The flowchart for patient selection is shown in **Figure 1**. There were 357 consecutive cases with AML who underwent their first MSD-HSCT or auto-HSCT between January 2014 and September 2021 in the Institute of Hematology, Chinese Academy of Medical Sciences. Patients with poor-risk AML, secondary AML, and acute promyelocytic leukemia (APL) were excluded from the study. It should be noted that disease risk could not be accurately classified in 23 (54.8%) out of 42 patients who received auto-HSCT because of the limited panel of gene mutations analyzed, meaning that these patients may carry high-risk gene mutations and classified as adverse-risk AML according to ELN2022. To control for imbalances in disease characteristics among the groups, propensity score matching was used among MSD-HSCT and auto-HSCT. There were 37 consecutive cases with *de novo* non-APL AML receiving chemotherapy as post-remission therapy between August 2019 and March 2023 in the Department of Hematology, Tianjin Union Medical Center of Nankai University. Patients in the chemotherapy group who relapsed or died within three cycles of consolidation chemotherapy were excluded because the patients in the HSCT group were in the status of CR1 with a median of three cycles of consolidation chemotherapy before HSCT. This study was approved by the Institutional Review Board and conducted in accordance with the Declaration of Helsinki. All the patients included in this study provided informed consent for their data to be used for research purposes.

### 2.2 Endpoints definitions and statistical analysis

CR was characterized by the presence of fewer than 5% blasts in the bone marrow and the absence of blasts in peripheral blood with no evidence of extramedullary leukemia. Relapse was defined as blasts ≥5% in bone marrow aspirations, the appearance of pathological blasts in peripheral blood, or extramedullary recurrence. OS was calculated from the start of induction chemotherapy to death or the last follow-up. DFS was defined as survival without relapse, while non-relapse mortality (NRM) was defined as death without previous relapse. Diagnosis and clinical staging of acute GVHD was based on the MAGIC criteria (9).

Induction chemotherapy cycles, consolidation chemotherapy cycles, and MRD status before HSCT were included in propensity score matching with a caliper width of 0.2. Chi-square test or Fisher’s exact test was used for categorical variables and Kruskal–Wallis test was used for continuous variables. Kaplan–Meier method with log-rank test was used for survival analysis. The cumulative incidence of competing risk outcomes was estimated using the Fine–Gray model. The Cox proportional hazard regression and Fine–Gray competing risks regression model were used for survival outcomes and competing risk outcomes to calculate hazard ratio (HR), respectively. Two-sided p-values were obtained from the regression model using Wald test. p-values<0.05 were considered statistically significant. All statistical analyses were performed using R version 4.0.5.

## 3 Results

### 3.1 Patient characteristics

After applying propensity score matching, 42 patients with favorable- and intermediate-risk *de novo* AML in CR1 before HSCT were included in each of the MSD-HSCT and auto-HSCT groups. Patients in the chemotherapy group who relapsed or died within three cycles of consolidation chemotherapy were excluded to reduce selection bias, regarding patients in the HSCT group who were in CR1 and received a median of three cycles of consolidation therapy before HSCT. A total of 27 patients receiving chemotherapy as post-remission therapy were included in the chemotherapy group. A total of 21 (50%), 16 (38.1%), and 12 (44.4%) patients had intermediate-risk AML in the auto-HSCT, MSD-HSCT, and chemotherapy group, respectively (p=0.547). There were 39 (92.9%), 41 (97.6%), and 24 (88.9%) patients who achieved CR1 with one cycle of induction chemotherapy in the three groups, respectively (p=0.377). The median follow-up days were 2,173.50 (IQR, 1,119.75, 2,707.50), 1,294.00 (IQR, 986.50, 2,306.25), and 1,019.00 (IQR, 538.00, 1,296.00) in the auto-HSCT, MSD-HSCT, and chemotherapy group, respectively (p<0.001). The patients’ characteristics are shown in **Table 1**.

**Table 1:** Patients’ characteristics.

	Overall (n=111)	Auto-HSCT (n=42)	MSD-HSCT (n=42)	Chemotherapy (n=27)	p-value
**Patient age, median (IQR)**	40.00 (29.50, 48.50)	33.00 (23.25, 46.25)	42.50 (31.25, 47.00)	39.00 (37.50, 49.50)	0.015
Patient gender, n (%)					
Male	68 (61.3)	30 (71.4)	22 (52.4)	16 (59.3)	0.195
Female	43 (38.7)	12 (28.6)	20 (47.6)	11 (40.7)	
ELN 2022, n (%)					
Favorable risk	62 (55.9)	21 (50.0)	26 (61.9)	15 (55.6)	0.547
Intermediate risk	49 (44.1)	21 (50.0)	16 (38.1)	12 (44.4)	
Induction cycle(s), n (%)					
1	104 (93.7)	39 (92.9)	41 (97.6)	24 (88.9)	0.377
2	7 (6.3)	3 (7.1)	1 (2.4)	3 (11.1)	
**Consolidation cycle, median (IQR)**	3.00 (3.00, 4.75)	3.00 (3.00, 3.00)	3.00 (3.00, 3.00)	5.00 (4.00, 5.00)	<0.001
MRD status after the first and the second cycle of chemotherapy, n (%)					
uMRD/uMRD	78 (70.3)	28 (66.7)	32 (76.2)	18 (66.7)	0.474
dMRD/uMRD	15 (13.5)	5 (11.9)	5 (11.9)	5 (18.5)	
uMRD/dMRD	3 (2.7)	0 (0.0)	1 (2.4)	2 (7.4)	
dMRD/dMRD	8 (7.2)	5 (11.9)	2 (4.8)	1 (3.7)	
NA	7 (6.3)	4 (9.5)	2 (4.8)	1 (3.7)	
Subtypes, n (%)					
t(8;21)(q22;q22.1)	21 (18.9)	6 (14.3)	11 (26.2)	4 (14.8)	0.533
inv(16)(p13.1q22) or t(16;16) (p13.1;q22)	11 (9.9)	4 (9.5)	3 (7.1)	4 (14.8)	
Others	79 (71.2)	32 (76.2)	28 (66.7)	19 (70.4)	
**Follow-up days, median (IQR)**	1,329.00 (972.50, 2,297.50)	2,173.50 (1,119.75, 2,707.50)	1,294.00 (986.50, 2,306.25)	1,019.00 (538.00, 1,296.00)	<0.001

IQR, interquartile range; ELN, European LeukemiaNet; MRD, measurable residual disease; uMRD, undetectable MRD; dMRD, detectable MRD; Auto-HSCT, autologous hematopoietic stem cell transplantation; MSD-HSCT, matched sibling donor hematopoietic stem cell transplantation.

### 3.2 Outcome in the overall population

**Figure 2:**
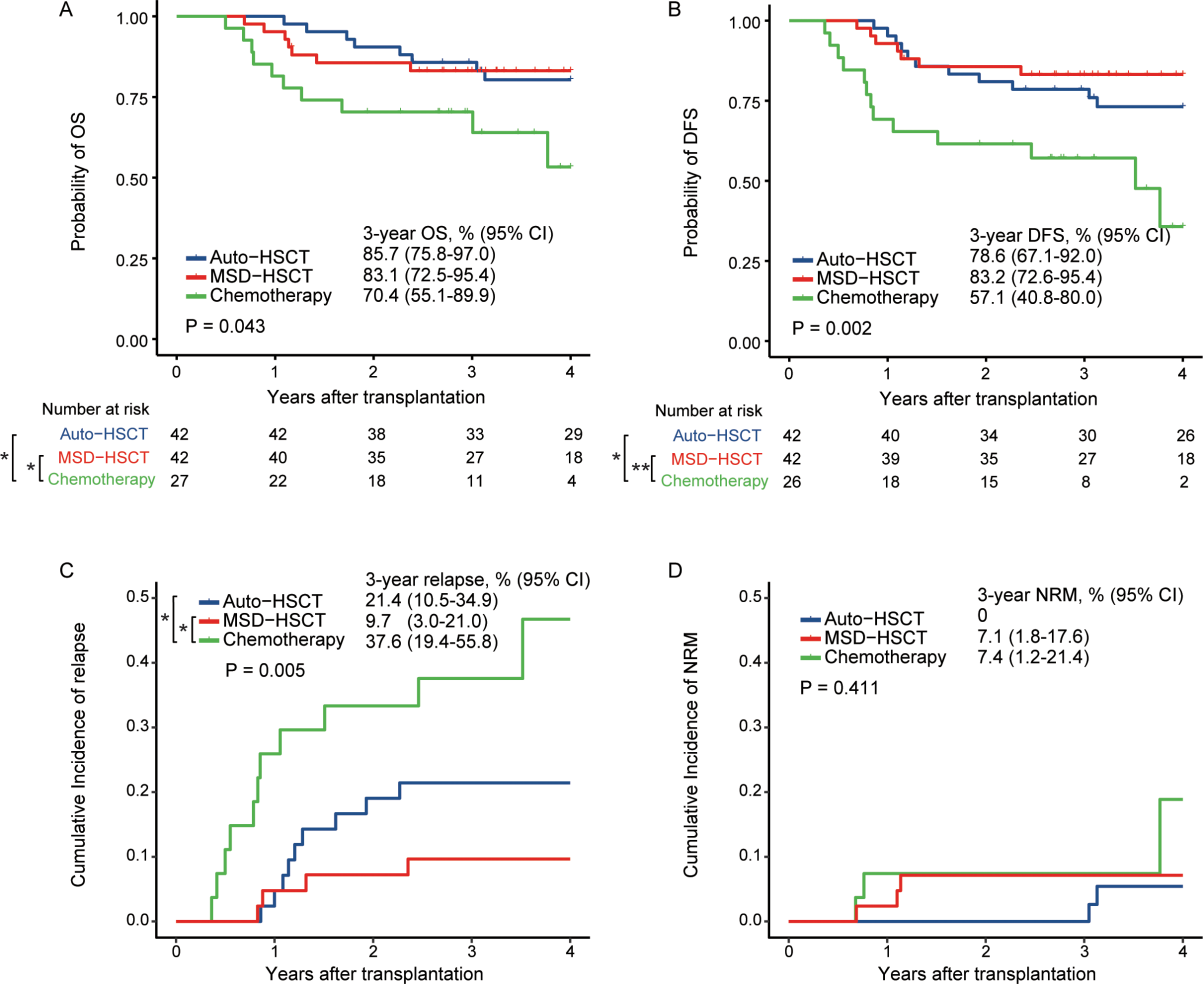
OS **(A)**, DFS **(B)**, RR **(C)**, and NRM **(D)** in the global population (*p < 0.05; **p < 0.01).

In the global population, the probability of 3-year OS was 85.7% (95% CI, 75.8%–97.0%), 83.1% (95% CI, 72.5%–95.4%), and 70.4% (95% CI, 55.1%–89.9%) in the auto-HSCT, MSD-HSCT, and chemotherapy group, respectively (**Figure 2A**, p=0.043). The 3-year DFS rate was 78.6% (95% CI, 67.1%–92.0%), 83.2% (95% CI, 72.6%–95.4%), and 57.1% (95% CI, 40.8%–80.0%), respectively (**Figure 2B**, p=0.002). The auto-HSCT and MSD-HSCT group had comparable OS (MSD-HSCT vs. auto-HSCT: HR=0.96; 95% CI, 0.35–2.66; p=0.943) and DFS (MSD-HSCT vs auto-HSCT: HR=0.66; 95% CI, 0.26–1.71; p=0.392). Compared to the chemotherapy group, OS was significantly improved in both the auto-HSCT group (HR=0.38; 95% CI, 0.15–0.96; p=0.042) and the MSD-HSCT group (HR=0.37; 95% CI, 0.14–0.96; p=0.041). Additionally, DFS was also significantly higher in auto-HSCT (HR, 0.36; 95% CI, 0.16–0.82, p=0.014) and MSD-HSCT (HR, 0.24; 95% CI, 0.10–0.60, p=0.002). We also calculated the power for the comparison between the auto-HSCT and MSD-HSCT groups, which showed comparable OS (auto-HSCT vs. MSD-HSCT: 85.7% vs. 83.1%; power = 0.062) and DFS (auto-HSCT vs. MSD-HSCT: 78.6% vs. 82.3%; power = 0.084), suggesting that auto-HSCT and MSD-HSCT may provide similar clinical benefits.

The cumulative incidence of 3-year relapse was significantly lower in patients who underwent auto-HSCT (21.4%, 95% CI 10.5%–34.9%; HR=0.41, 95% CI 0.17–0.99; p=0.049) and MSD-HSCT (9.7%, 95% CI 3.0%–21.0%; HR=0.18, 95% CI, 0.06–0.57; p=0.003) than that in patients who received chemotherapy (37.6%; 95% CI, 19.4%–55.8%; **Figure 2C**). The 3-year cumulative incidence of NRM was 0.0%, 7.1% (95% CI, 1.8%–17.6%), and 7.4% (95% CI, 1.2%–21.4%) in the auto-HSCT, MSD-HSCT, and chemotherapy group, respectively (p=0.411, **Figure 2D**).

### 3.3 Analysis of factors affecting DFS in the overall population

Factors affecting DFS were analyzed in the global patients (**Table 2**). In univariate analysis, both intermediate risk (HR=2.57; 95% CI, 1.23–5.37; p=0.012), FLT3-ITD mutation (HR=2.16; 95% CI, 0.96–4.83; p=0.062), not achieving uMRD within the first two cycles of chemotherapy (HR=2.26; 95% CI, 1.12–4.60, p=0.024), and receiving chemotherapy as post-remission treatment (HR=2.75; 95% CI, 1.23–6.18; p=0.014) were factors associated with decreased probability of DFS. Considering that patients with intermediate-risk AML and FLT3-ITD mutations are independent covariables, we incorporated disease risk into multivariable analysis. The results indicated that chemotherapy was an independent adverse prognostic factor of DFS in comparison to auto-HSCT (HR=2.82; 95% CI, 1.26–6.32, p=0.012), while DFS was similar between the MSD-HSCT and auto-HSCT groups (HR=0.80; 95% CI, 0.31–2.09, p=0.645).

**Table 2:** Analysis of factors affecting disease-free survival.

Variables	Univariable Analysis		Multivariable analysis	
	HR (95% CI)	p-value	HR (95% CI)	p-value
Patient age				
<35 years	Reference			
≥35 years	1.67 (0.77–3.63)	0.195		
ELN 2022				
Favorable risk	Reference		Reference	
Intermediate risk	2.57 (1.23–5.37)	0.012	2.22 (1.04–4.75)	0.039
FLT3-ITD				
Without FLT3-ITD mutation	Reference			
With FLT3-ITD mutation	2.16 (0.96–4.83)	0.062		
Induction cycle(s)				
1	Reference			
2	1.93 (0.59–6.37)	0.278		
MRD status				
MRD-/MRD-*	Reference		Reference	
Others	2.26 (1.12–4.6)	0.024	1.83 (0.88–3.8)	0.108
Treatment				
Auto-HSCT	Reference		Reference	
MSD-HSCT	0.66 (0.26–1.71)	0.392	0.80 (0.31–2.09)	0.645
Chemotherapy	2.75 (1.23–6.18)	0.014	2.82 (1.26–6.32)	0.012

*MRD-/MRD-, undetectable MRD after the first and the second cycle of chemotherapy.

HR, hazard ratio; MRD, measurable residual disease; uMRD, undetectable MRD; Auto-HSCT, autologous hematopoietic stem cell transplantation; MSD-HSCT, matched sibling donor hematopoietic stem cell transplantation.

### 3.4 Outcome by disease risk

**Figure 3:**
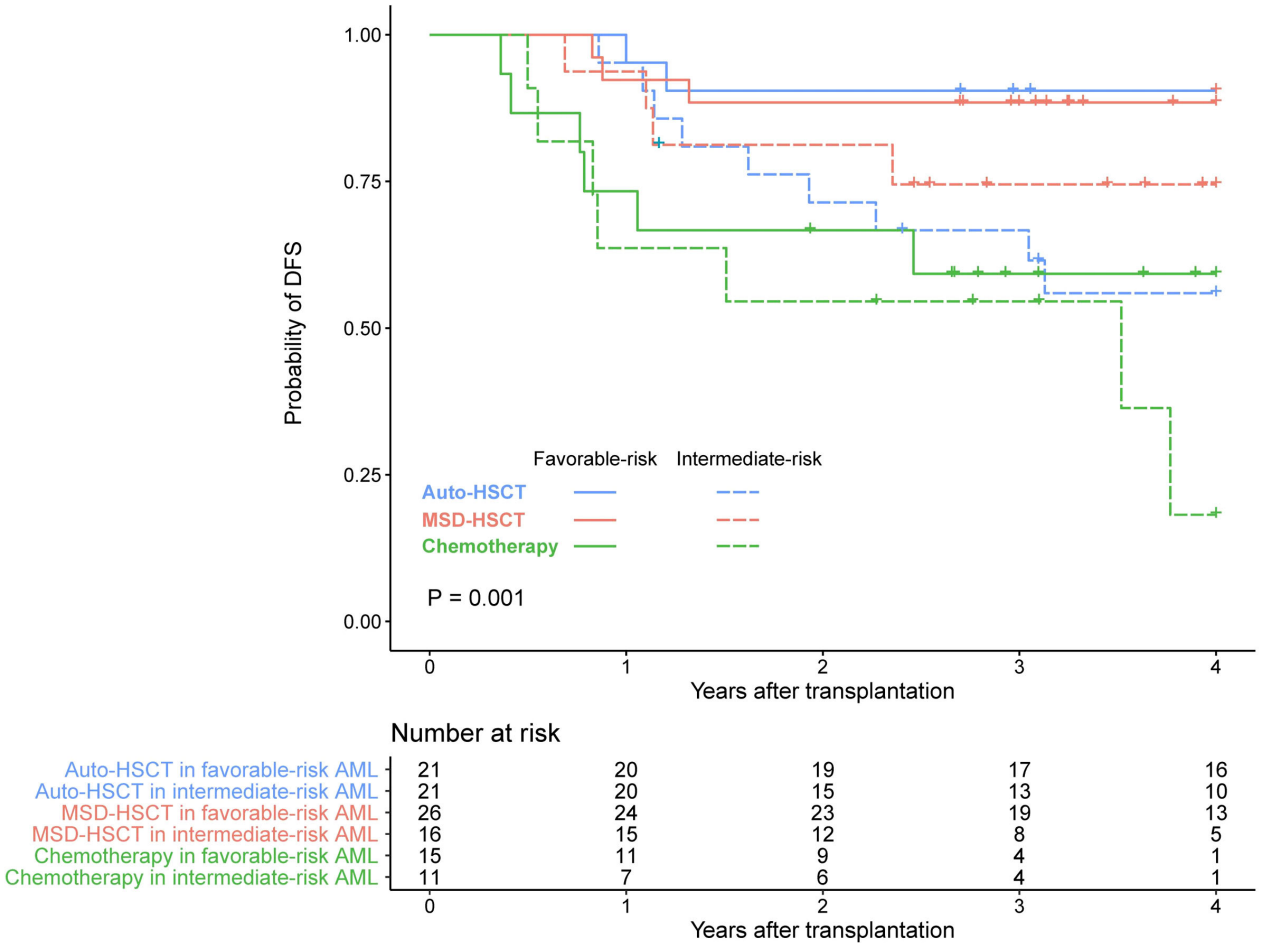
DFS in patients with favorable- or intermediate-risk AML receiving auto-HSCT, MSD-HSCT, or chemotherapy.

There were 62 patients with favorable-risk AML and 49 patients with intermediate-risk AML. Two out of 16 patients in the MSD group with FLT3-ITD mutations allelic ratio > 0.5. We compared the survival of different post-remission treatments according to ELN 2022 disease risk. DFS regarding disease risks and different treatments is shown in **Figure 3**.

**Figure 4:**
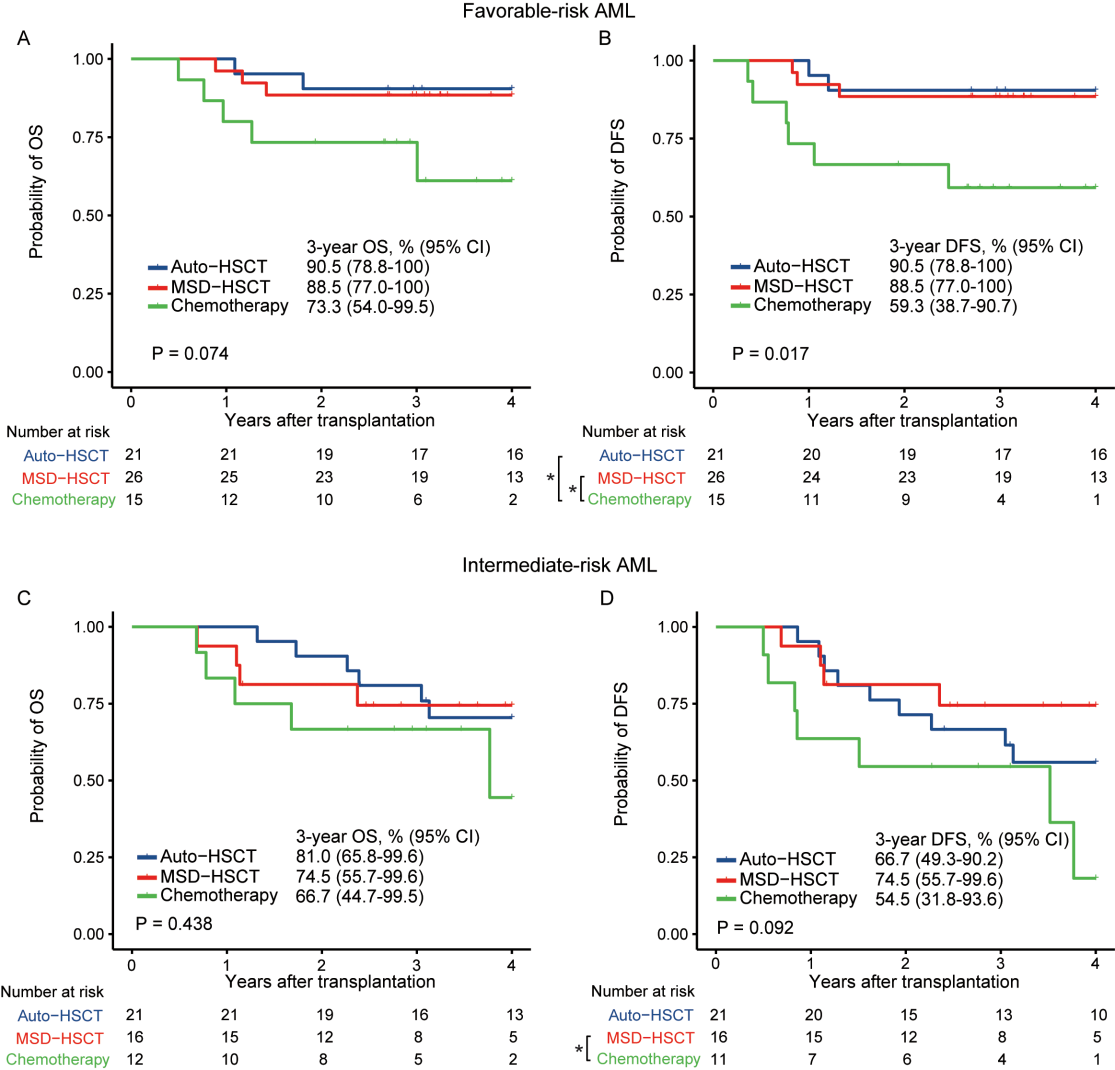
OS and DFS in patients with favorable-risk **(A, B)** and intermediate-risk **(C, D)** AML (*p < 0.05).

For a further detailed comparison, in patients with favorable-risk AML, there was a trend of higher OS in patients who received auto-HSCT (HR=0.23; 95% CI, 0.04–1.19; p=0.080) and MSD-HSCT (HR=0.29; 95% CI, 0.07–1.21; p=0.089) compared to those undergoing chemotherapy. The probability of 3-year OS was 90.5% (95% CI, 78.8%–100%), 88.5% (95% CI, 77.0%–100%), and 73.3% (95% CI, 54.0%–99.5%) in the auto-HSCT, MSD-HSCT, and chemotherapy group, respectively (**Figure 4A**, p=0.074). As for DFS, both auto-HSCT (HR=0.19; 95% CI, 0.04–0.92; p=0.040) and MSD-HSCT (HR=0.23; 95% CI, 0.06–0.92; p=0.038) were associated with significantly improved DFS as compared to chemotherapy. The probability of 3-year DFS in patients with favorable-risk AML was 90.5% (95% CI, 78.8%–100%), 88.5% (95% CI, 77.0%–100%), and 59.3% (95% CI, 38.7%–90.7%), respectively (**Figure 4B**, p=0.017).

In patients with intermediate-risk AML, there was no significant difference in OS among the auto-HSCT, MSD-HSCT, and chemotherapy group, with a 3-year OS of 81.0% (95% CI, 65.8%–99.6%), 74.5% (95% CI, 55.7%–99.6%), and 66.7% (95% CI, 44.7%–99.5%), respectively (**Figure 4C**, p=0.438). DFS was significantly greater after receiving MSD-HSCT compared to those who received chemotherapy (HR=0.29; 95% CI, 0.08–0.99; p=0.047). However, DFS between MSD-HSCT and auto-HSCT was similar (HR=0.62; 95% CI, 0.19–2.00, p=0.419. The probability of 3-year DFS in patients with intermediate-risk AML was 66.7% (95% CI, 49.3%–90.2%), 74.5% (95% CI, 55.7%–99.6%), and 54.5% (95% CI, 31.8%–93.6%) in the auto-HSCT, MSD-HSCT, and chemotherapy group (**Figure 4D**, p=0.092).

## 4 Discussion

Currently, after remission of AML patients with induction chemotherapy, post-remission treatment is selected according to 2022 ELN risk stratification at onset and MRD dynamic monitoring. For favorable-risk AML in CR1, the guidelines recommend consolidation chemotherapy or ASCT, while the recommendations were uncertain for intermediate-risk AML in CR1 (8).

In our study, we explored optimal consolidation strategies after CR according to risk stratification and MRD for AML patients with favorable- and intermediate-risk AML. We found that the 3-year DFS was 78.6%, 83.2%, and 57.1% in the auto-HSCT, MSD-HSCT, and chemotherapy groups, respectively (p=0.002). Based on further analysis according to the 2022 ELN disease risk, the 3-year DFS was 90.5%, 88.5%, and 59.3% in the auto-HSCT, MSD-HSCT, and chemotherapy group, respectively (p=0.017) in patients with favorable-risk AML, while it was 66.7%, 74.5%, and 54.5%, respectively (p=0.092) in patients with intermediate-risk AML. Both auto-HSCT and MSD-HSCT were associated with significantly better DFS as compared to chemotherapy in patients with favorable-risk AML.

Previous studies have either supported this result or come to different results, due to differences of patient’s information. A long-term follow-up of the EORTC/GIMEMA AML-8A study also confirms a longer DFS with allo-bone marrow transplantation (BMT) or auto-BMT when compared to chemotherapy in younger AML patients in CR1 (10), while the study by Limvorapitak et al. supported the preference for MSD-HSCT in patients with intermediate-risk AML patients, and there were no survival differences between auto-HSCT and chemotherapy (11). Many studies suggested patients with intermediate-risk AML who underwent MSD-HSCT in CR1 had the best outcomes (6, 12). A meta-analysis by Koreth concluded that allo-HSCT had significant RFS and OS benefit for intermediate- and for poor-risk AML but not for favorable-risk AML patients in CR1 (4). Several studies also confirmed the advantages of auto-HSCT (7, 13–16). A retrospective, multicenter analysis supported intermediate-risk AML patients with no FLT3-ITD, and no detectable MRD can be offered ASCT as a therapeutic option compared with to haploidentical donor HSCT (17). One study by Yegin reported 101 AML patients in CR1 who were not eligible for allo-HSCT and also confirmed better DFS in auto-HSCT recipients compared to chemotherapy arm (43% vs. 4.8%, p=0.008) (18). A present meta-analysis indicated that AML patients in CR1 receiving auto-HSCT had higher DFS and lower relapse compared to chemotherapy treatment (19).

In univariate analysis, intermediate risk, FLT3-ITD mutation, not achieving uMRD within the first two cycles of chemotherapy, and receiving chemotherapy as post-remission treatment were factors associated with decreased probability of DFS. Recent studies also used MRD to guide treatment strategies in AML patients (20, 21). FLT3–ITD had been shown to be associated with increased risk of relapse and a worse prognosis (22–25).

This study has several limitations. First, this was a retrospective study. A retrospective study could not achieve strict randomization and may be affected by case selection bias. Second, in these subgroups, results should be explained with caution due to small numbers. Further studies with larger cohorts and prospective designs are needed to validate these findings.

## 5 Conclusion

In conclusion, MSD-HSCT or auto-HSCT is recommended for favorable- and intermediate-risk AML patients, and the efficacy is better than chemotherapy. There is a statistical difference in the favorable-risk group while a trend in DFS for intermediate-risk cohorts. Further studies are required to confirm the outcomes.

## Data Availability

The original contributions presented in the study are included in the article. Further inquiries can be directed to the corresponding authors.
